# *Aenictus hoelldobleri* sp. n., a new species of the *Aenictus ceylonicus* group (Hymenoptera, Formicidae) from China, with a key to the Chinese members of the group

**DOI:** 10.3897/zookeys.516.9927

**Published:** 2015-08-10

**Authors:** Michael Staab

**Affiliations:** 1 Chair of Nature Conservation and Landscape Ecology, Institute of Earth and Environmental Sciences, University of Freiburg, Tennenbacherstraße 4, 79106 Freiburg, Germany

**Keywords:** Army ants, Dorylinae, Gutianshan National Nature Reserve, species descriptions, subtropical forest, taxonomy

## Abstract

*Aenictus* is the most species-rich genus of army ants in the subfamily Dorylinae and one of the most species rich ant genera in China and the world. In this paper, a new species of the *Aenictus ceylonicus* group, *Aenictus hoelldobleri* **sp. n.**, is described and illustrated based on the worker caste. The new species occurs in the subtropical forests of south-east China and is morphologically most similar to *Aenictus henanensis* Li & Wang, 2005 and *Aenictus wudangshanensis* Wang, 2006. *Aenictus hoelldobleri* **sp. n.** can be distinguished from both species by the shape of the subpetiolar process. The new species also resembles *Aenictus lifuiae* Terayama 1984 and *Aenictus thailandianus* Terayama & Kubota, 1993 but clearly differs in various features of the cuticular sculpture. A key to the Chinese species of the *Aenictus ceylonicus* group based on the worker caste is provided, which may help to reassess and clarify the taxonomic status of the abundant Chinese records of the true *Aenictus ceylonicus* (Mayr, 1866), a species which almost certainly does not occur in China. Several new locality records are given, among them the first record of *Aenictus watanasiti* Jaitrong & Yamane, 2013 from China.

## Introduction

Army ants form a monophyletic group in the subfamily Dorylinae (Brady et al. 2014). As a taxonomic group, all army ants can be characterized by a set of ecological and behavioral traits, most notably the specialized queen morphology, frequent nest relocations, and mass foraging raids for arthropod prey (Gotwald 1995, Kronauer 2009). Recently, Brady et al. (2014) clarified the in-depth phylogeny of army ants and their related taxa. Nevertheless, the species level taxonomy of most army ants is still far from being resolved and new species continue to be described (e.g. Bharti et al. 2012, Jaitrong and Yamane 2013, Staab 2014a, Liu et al. 2015b).

Of all army ant genera the genus *Aenictus* is the most species rich and widely distributed. Currently, 181 valid species (AntCat 2015) are known from the Mediterranean and the tropical and subtropical regions of Asia, Africa, and Australia (Gotwald 1995). As far as it is known, almost all *Aenictus* species are specialized predators of other ants, which are captured by raiding nests (e.g. Hirosawa et al. 2000, Hashimoto and Yamane 2014), but a few species are more generalized predators of arthropods (Schneirla and Reyes 1966) or can even occasionally be trophobiotic (Staab 2014b).

Over the last years, in a series of significant papers Weeyawat Jaitrong, Seiki Yamane, and co-workers divided the south-east Asian *Aenictus* fauna in 12 species groups based on the worker caste (Jaitrong and Yamane 2011, the key to species groups is freely available online at http://www.antwiki.org/wiki/Key_to_Aenictus_species_groups), which the authors comprehensively revised (Jaitrong and Yamane 2010, Jaitrong et al. 2010, Jaitrong and Yamane 2011, Wiwatwitaya and Jaitrong 2011, Jaitrong and Hashimoto 2012, Jaitrong and Yamane 2012, Jaitrong and Wiwatwitaya 2013, Jaitrong and Yamane 2013). Workers of the *Aenictus ceylonicus* species group can be distinguished from all other species groups by the linear mandibles, the presence of a gap between the closed mandibles and the anterior clypeal margin, and an anterior clypeal margin without denticles (Jaitrong and Yamane 2011, 2013). Out of all *Aenictus* species groups the *Aenictus ceylonicus* group is most diverse. In their comprehensive revision Jaitrong and Yamane (2013) treat 23 worker-based species from south-east Asia, of which 19 were newly described. Recently, Liu et al. (2015b) added a further new species from tropical China and gave new species records for the south-west Chinese fauna (Liu et al. 2015a). Despite this, it is likely that several species still await discovery and description in this region.

In the present paper *Aenictus hoelldobleri* Staab sp. n. is described, a new species of the *Aenictus ceylonicus* group from the subtropical forests of south-east China. Furthermore, the first Chinese record of *Aenictus watanasiti* Jaitrong & Yamane, 2013 is reported, and new Chinese locality records for *Aenictus formosensis* Forel, 1913, *Aenictus fuchuanensis* Zhou, 2001, *Aenictus thailandianus* Terayama & Kubota, 1993, and *Aenictus wudangshanensis* Wang, 2006 are added. As the key from Jaitrong and Yamane (2013) did not include a few *Aenictus ceylonicus* group species that have been described from non-tropical China, an updated key to the ten *Aenictus ceylonicus* group species known from China is provided, based on the worker caste.

## Methods

All morphological observations were made with a Leica SD6 stereomicroscope, which was equipped with an ocular micrometer to take measurements. Automontage images of specimens were provided by http://www.antweb.org/ (photographer: Michele Esposito) or extracted from Jaitrong and Yamane (2013) and Liu et al. (2015b).

The general worker terminology as well as abbreviations used for measurements and indices follow Jaitrong and Yamane (2011, 2013). All measurements are expressed in millimeters and are:

CI Cephalic index, HW / HL × 100.

HL Maximum head length in full-face view, measured from the midpoint of the anterior clypeal margin to the midpoint of the posterior margin of the head.

HW Maximum head width in full-face view.

ML Mesosomal length measured from the point at which the pronotum meets the cervical shield to the posterior base of the metapleuron in profile.

PL Petiole length measured from the anterior margin of the peduncle to the posteriormost point of the petiolar tergite in profile.

SI Scape index: SL / HW × 100.

SL Scape length excluding the basal constriction and condylar bulb.

TL Total length, measured roughly from the anterior margin of head to the tip of gaster in fully stretched specimens in profile.

### Depositories of type material

CASC California Academy of Science Collection, San Francisco, California, USA.

HLMD Hessisches Landesmuseum Darmstadt, Darmstadt, Germany.

IZAS Insect Collection of the Institute of Zoology, Chinese Academy of Sciences, Beijing, China.

ZMBH Museum für Naturkunde, Berlin, Germany.

### Distribution maps

Distribution maps for all Chinese *Aenictus ceylonicus* group species were composed from the locality records given in the original descriptions, the records presented in this paper and the records listed in Jaitrong and Yamane (2013) and Liu et al. (2015a). Maps were created by manually adding species localities with the graphical software GIMP 2 (http://www.gimp.org) on a map extracted from the R-package “OpenStreetMap” (http://cran.r-project.org/web/packages/OpenStreetMap). The numerous Chinese records of *Aenictus ceylonicus* (Mayr, 1866) compiled by Guénard and Dunn 2012 were not taken into account, as this species likely does not occur in China (see Discussion for a detailed explanation).

## Results Systematics

### *Aenictus ceylonicus* species group

**Diagnosis.** Jaitrong and Yamane (2011) defined this species group as follows:

*Antenna 10-segmented; scape reaching or extending beyond half of head length, but not reaching the occipital corner of head in full-face view. Mandible linear; its basal and lateral margins almost parallel; masticatory margin with large apical tooth followed by medium-sized subapical tooth; between subapical tooth and basal tooth 0–6 small denticles present. With mandibles closed, a gap present between mandibles and anterior margin of clypeus. Anterior clypeal margin weakly concave or almost straight, lacking denticles. Frontal carina short and thin, reaching or slightly extending beyond the level of posterior margin of torulus; anterior curved extension of frontal carina reaching or extending beyond the level of anterior clypeal margin in full-face view; parafrontal ridge absent. Promesonotum usually convex dorsally and sloping gradually to propodeum. Subpetiolar process developed. Head and first gastral tergite smooth and shiny. Body yellowish, reddish or dark brown; typhlatta spot absent*.

**Remarks.** The *Aenictus ceylonicus* group can be easily distinguished from other *Aenictus* species groups by the combination of linear mandibles, the presence of a gap between the closed mandibles and the anterior clypeal margin, and an almost straight or feebly concave anterior clypeal margin, which lacks denticles.

### Synoptic species list of *Aenictus ceylonicus* group species known from China:

*Aenictus formosensis* Forel, 1913 (Taiwan, Zhejiang)

*Aenictus fuchuanensis* Zhou, 2001 (Guangxi, Hong Kong, Jiangxi)

*Aenictus henanensis* Li & Wang, 2005 (Henan)

*Aenictus hoelldobleri* sp. n. (Jiangxi, Zhejiang)

*Aenictus lifuiae* Terayama, 1984 (Taiwan)

*Aenictus maneerati* Jaitrong & Yamane, 2013 (Yunnan)

*Aenictus thailandianus* Terayama & Kubota, 1993 (Yunnan, Guizhou)

*Aenictus watanasiti* Jaitrong & Yamane, 2013 (Guizhou)

*Aenictus wudangshanensis* Wang, 2006 (Hubei, Zhejiang)

*Aenictus yangi* Liu, Hita Garcia, Peng & Economo, 2015 (Yunnan)

### Key to Chinese *Aenictus ceylonicus* group species

Key to Chinese *Aenictus ceylonicus* group species based on the worker caste, modified and updated after the key of Jaitrong and Yamane (2013), which is freely available online at http://www.antwiki.org/wiki/Key_to_southeastern_Asian_Aenictus_ceylonicus_group_species and the extension of this key by Liu et al. (2015b):

**Table:** 

1	Mandible with 2-6 teeth/denticles between subapical and basal teeth (mandible with more than 4 teeth/denticles) (Fig. 1A, B)	**2**
–	Mandible with 0-1 tooth/denticle between subapical and basal teeth (mandible with 3-4 teeth/denticles) (Fig. 1C, D)	**7**
2	Promesonotum entirely punctate (Fig. 4A), at most lateral face of pronotum partly smooth and shiny; dorsum of postpetiole punctate (Fig. 3A) (Guizhou)	***Aenictus thailandianus* Terayama & Kubota**
–	Promesonotum predominantly smooth and shiny (Fig. 4B, C, D); dorsum of postpetiole smooth and shiny (Figs 3B, 3C, 3D)	**3**
3	Subpetiolar process weakly developed, low and rounded, not rectangular (Fig. 4B)	**4**
–	Subpetiolar process well developed and rectangular (Figs 4C, 4D)	**5**
4	Dorsum of propodeum straight in profile, entirely microreticulate and opaque; promesonotum microreticulate except posterior half of pronotum smooth and shiny; masticatory margin of mandible with large apical tooth, followed by a small preapical tooth, and 5 minute denticles (Henan)	***Aenictus henanensis* Li & Wang**
–	Dorsum of propodeum weakly convex to almost straight in profile, punctate but somewhat shiny; promesonotum entirely smooth and shiny except for reticulate anteriormost portion (Fig. 3B); masticatory margin of mandible with large apical tooth followed by a series of 6-7 denticles of two sizes, the larger alternating with 1-2 smaller (Fig. 1B) (Taiwan)	***Aenictus lifuiae* Terayama**
5	Dorsum of mesonotum and petiolus entirely smooth and shiny (Fig. 3C)	***Aenictus yangi* Liu, Hita Garcia, Peng & Economo**
–	Dorsum of mesonotum and petiolus finely reticulate (Fig. 3D)	**6**
6	Subpetiolar process rectangular-trapezoidal, its ventral outline with a thin almost transparent lamellae (Fig. 4C); masticatory margin of mandible with 4 (rarely 3) denticles (total number of mandibular teeth 6-7, including apical, subapical and basal tooth) (Jiangxi, Zhejiang)	***Aenictus hoelldobleri* sp. n.**
–	Subpetiolar process rectangular, its apex very acute and directed downwards medially (Fig. 4D); masticatory margin of mandible with 6 denticles (total number of mandibular teeth 9 including apical, subapical and basal tooth) (Hubei, Zhejiang)	***Aenictus wudangshanensis* Wang**
7	Mandible with 3 teeth including apical and basal tooth (Fig. 1C) (Guizhou)	***Aenictus watanasiti* Jaitrong & Yamane**
–	Mandible with 4 teeth including apical and basal tooth (Fig. 1D)	**8**
8	Subpetiolar process well-developed, subrectangular with convex ventral lamella, and with anterior and posterior corners acutely or bluntly angulated (Fig. 4E); head longer than broad, at maximum as broad as long (CI 90-100) (Fig. 2B) (Taiwan, Zhejiang)	***Aenictus formosensis* Forel**
–	Subpetiolar process weakly developed or very low (Fig. 4F, G); head broader than long, at minimum as broad as long (CI 100-112) (Fig. 2C, D)	**9**
9	Subpetiolar process very low, with anterior and posterior denticles that protrude downwards (Fig. 4G); head in full-face view subrectangular, posterior margin feebly concave (Fig. 2D) (Yunnan)	***Aenictus maneerati* Jaitrong & Yamane**
–	Subpetiolar process weakly developed, in profile its ventral outline almost straight or weakly convex, without denticles (Fig. 4F); head in full face view not rectangular, posterior margin weakly convex or straight (Fig. 2C) (Guangxi, Hong Kong	***Aenictus fuchuanensis* Zhou**

**Figure 1.**
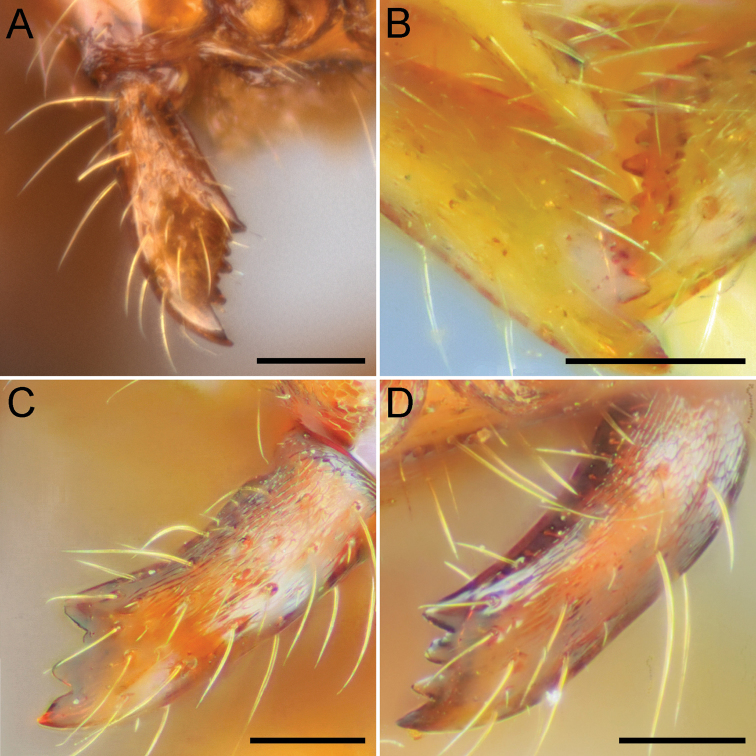
Mandible of Chinese *Aenictus ceylonicus* group species in full face view. **A** *Aenictus yangi* **B** *Aenictus lifuiae* **C** *Aenictus watanasiti* **D** *Aenictus maneerati*. Scale bars – 0.1 mm. Image **A** is from Liu et al. (2015b), all other images are from Jaitrong and Yamane (2013).

**Figure 2.**
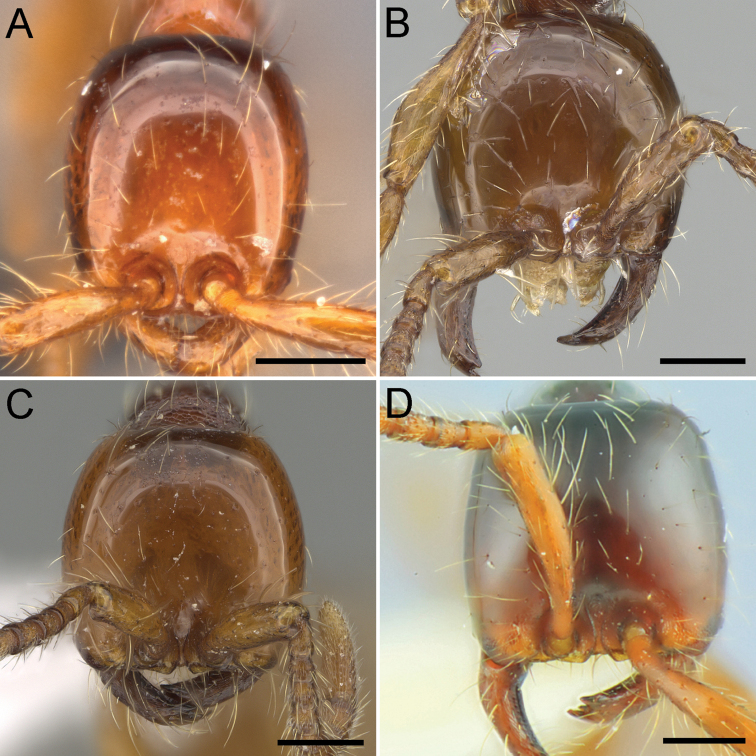
Head of Chinese *Aenictus ceylonicus* group species in full face view. **A** *Aenictus yangi* **B** *Aenictus formosensis* (CASENT0914926) **C** *Aenictus fuchuanensis* (CASENT0914926) **D** *Aenictus maneerati*. Scale bars – 0.2 mm. Image **A** is from Liu et al. (2015b), **B** and **C** are from http://www.antweb.org (photographer: Michele Esposito), and **D** is from Jaitrong and Yamane (2013).

**Figure 3.**
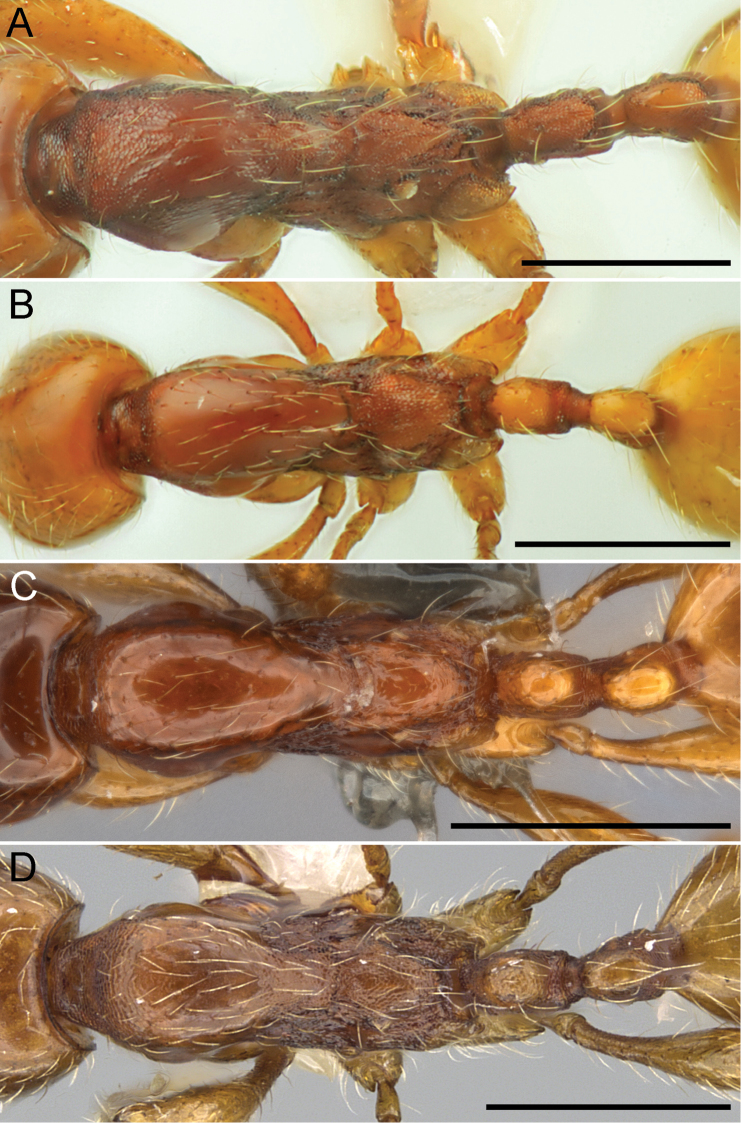
Mesosoma and waist segments of Chinese *Aenictus ceylonicus* group species in dorsal view. **A** *Aenictus thailandianus* **B** *Aenictus lifuiae* **C** *Aenictus yangi* **D** *Aenictus hoelldobleri* sp. n. (CASENT0914932).

**Figure 4.**
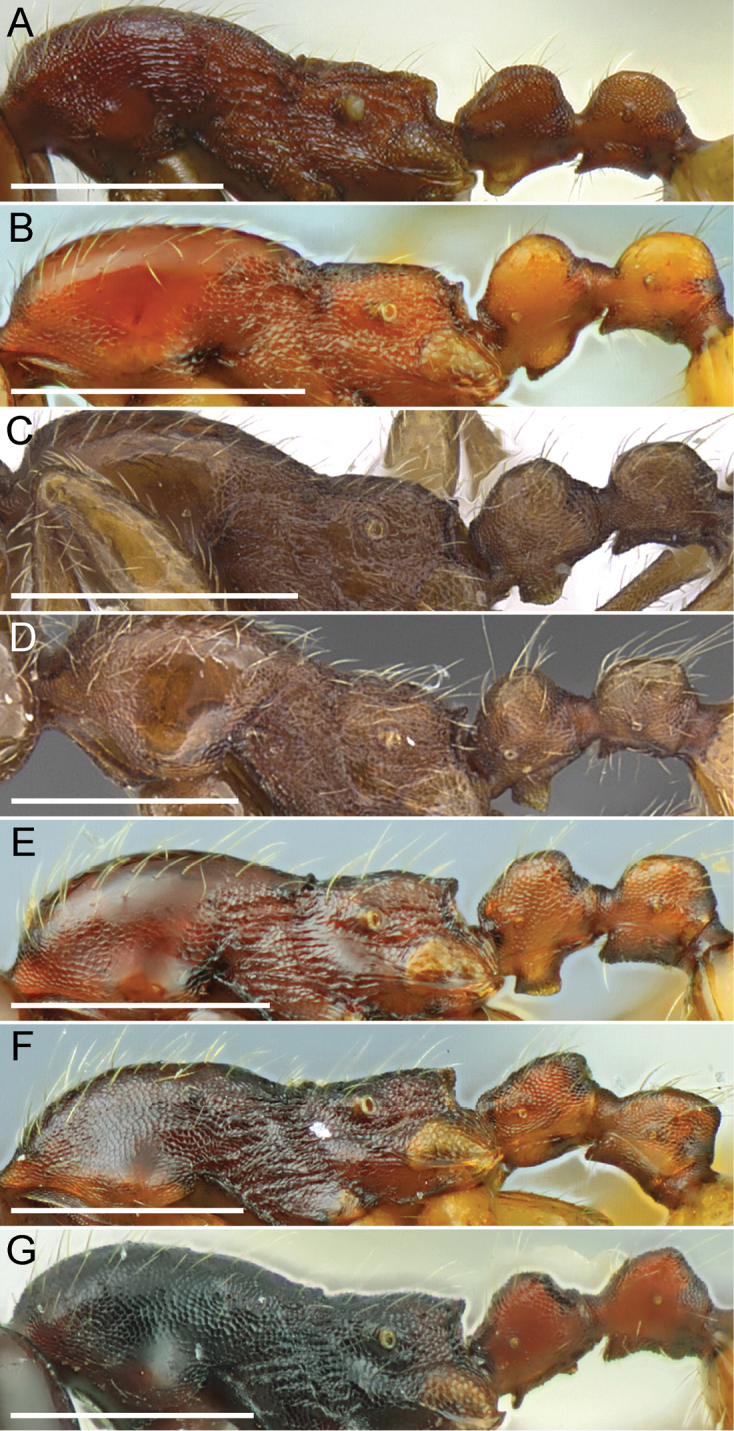
Mesosoma and waist segments of Chinese *Aenictus ceylonicus* group species in profile. **A** *Aenictus thailandianus* **B** *Aenictus lifuiae* **C** *Aenictus hoelldobleri* sp. n. (CASENT0914932) **D** *Aenictus wudangshanensis* (CASENT0914927) **E** *Aenictus formosensis* **F** *Aenictus fuchuanensis* **G** *Aenictus maneerati*. Scale bars – 0.5 mm. Images **C** and **D** are from http://www.antweb.org (photographer: Michele Esposito). All other images are from Jaitrong and Yamane (2013).

### Description of new species

#### Aenictus hoelldobleri

Taxon classificationAnimaliaHymenopteraFormicidae

Staab sp. n.

http://zoobank.org/8617546B-AAD7-43BF-9215-7BD1B161465E

Figs 3D , 4C , 5A–D

##### Holotype.

Worker from CHINA, Jiangxi Province, near the village Xingangshan, ca. 15 km SE of Wuyuan, 29°4'39"N / 117°55'20"E, 300 m asl, 6.VII.2013, hand collection on ground, leg. Michael Staab, label “MS1647”, deposited in IZAS.

##### Paratypes.

20 workers in total, all with the same data as holotype (3 in CASC: CASENT0914931, CASENT0914932, CASENT0914933, 4 in HLMD, 10 in IZAS, 3 in ZMBH).

##### Measurements and indices.

**Holotype**: TL 2.88, HL 0.65, HW 0.57, SL 0.46, ML 0.95, PL 0.25, CI 88, SI 81. **Paratypes** (n=20 measured): TL 2.34-2.88, HL 0.52-0.68, HW 0.48-0.60, SL 0.40-0.50, ML 0.83-1.02, PL 0.20-0.25, CI 84-92, SI 75-86.

##### Worker description

(holotype and paratypes). Head in full-face view slightly longer than broad (CI 84-92), sides slightly convex, posterior margin slightly rounded to almost straight, and occipital corners broadly rounded; occipital margin bearing distinct carina. Antennal scape relatively long (SI 75-86), extending well beyond 2/3 of head length but not reaching posterolateral corner of head; antennal segments II-VIII each broader than long, antennal segments IX and X longer than broad; length of segments II-IX continuously rising; terminal segment (X) longer than VIII and IX taken together; last four segments forming indistinct club. Frontal carina long and distinct, surpassing posterior margin of antennal torulus. Clypeus very short, its anterior margin almost straight to feebly concave, with lateral portions bluntly angled. Masticatory margin of mandible with large acute apical tooth, followed by medium-sized subapical tooth, 4 (rarely 3) small denticles, and medium-sized basal tooth; denticles and basal tooth worn out and hard to see in few paratypes; basal margin straight, lacking denticles. Gap between closed mandibles and anterior clypeal margin relatively small, about 0.5-0.6 times as broad as maximum width of mandible. With mesosoma in profile, promesonotum strongly convex, sloping gradually to the weakly developed but distinct metanotal groove; mesopleuron relatively short, demarcated from metapleuron by distinct groove; metapleural gland bulla moderately large, its maximum diameter about 1.3 times as long as distance between propodeal spiracle and most proximate part of metapleural gland bulla. Dorsal outline of propodeum in profile weakly convex, gently sloping posteriorly; propodeal junction angulated, overhanging declivity of propodeum, which is shallowly concave and encircled with thin but distinct rim. Petiole excluding subpetiolar process in profile slightly higher than long; petiolar node with steep anterior face and broadly convex dorsal outline; subpetiolar process developed, its ventral outline trapezoidal and rectangular, its apex on anterior part of process; ventralmost part of subpetiolar process with thin almost transparent lamellae. Postpetiole slightly shorter than petiole, in profile dorsal outline of node convex with small entirely flat area on dorsum; postpetiolar process developed, angulate, pointing anteriorly.

Head entirely smooth and shiny except for finely punctate antennal torulus. Mandible finely striate. Antennal scape entirely punctate. Mesosoma entirely finely reticulate with exception of pronotum and metapleuron; pronotum finely reticulate with large smooth and shiny median area on sides and dorsum; in few larger paratypes pronotal dorsum very finely and superficially reticulate but still smooth and shiny; anterior part of metapleuron smooth and shiny (with very fine and superficial longitudinal rugae in few larger paratypes). Entire petiole, including subpetiolar process, finely reticulate. Postpetiole finely reticulate, with flat surface on dorsum smooth and shiny. Gaster entirely smooth and shiny. Legs weakly punctate, more strongly so on tibiae, coxae smooth and shiny.

Body except sides of mesosoma with abundant standing and decumbent hairs of variable length; length of longest hairs on dorsum of head and pronotum 0.15–0.20 mm. Antennal scapes and legs with abundant decumbent hairs. Antennae, mesosoma, petiole and postpetiole reddish to yellowish brown, gaster and legs yellowish brown.

Male and female are unknown.

**Figure 5.**
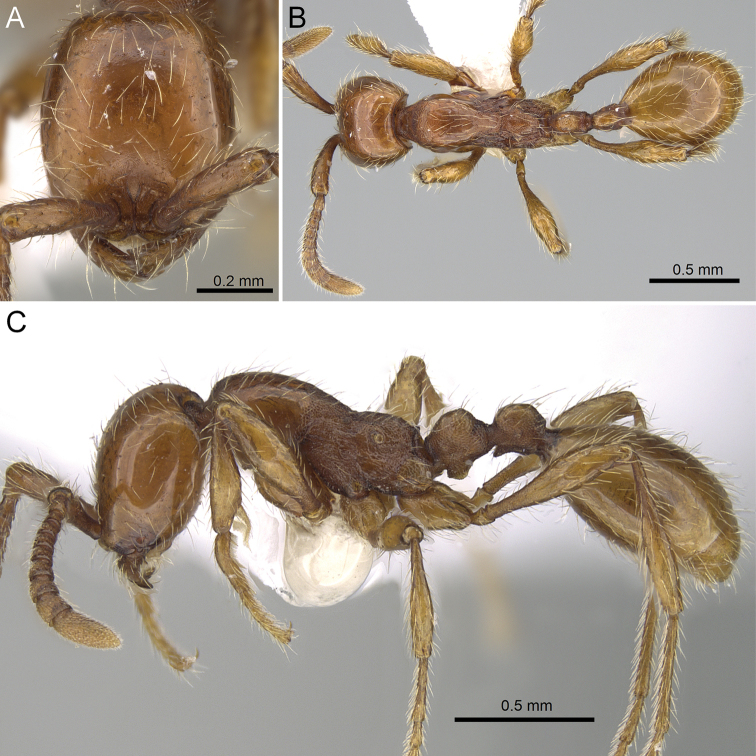
*Aenictus hoelldobleri* sp. n. (CASENT0914932). **A** Head in full face view **B** Body in profile **C** Body in dorsal view. All images are from http://www.antweb.org (photographer: Michele Esposito).

##### Etymology.

The species epithet is a patronym in honor of the great German myrmecologist Berthold ‘Bert’ Hölldobler and his invaluable and outstanding contributions to our understanding of ant societies.

##### Non-type material examined.

eight workers in total; two from CHINA, Zhejiang Province, Gutianshan National Nature Reserve, ca. 30 km NW of Kaihua, 29°12'2"N / 118°7'54"E, 345 m asl, 29.V.2009, pitfall trap, leg. Andreas Schuldt, label: “CSP25/NE4(2009)” (IZAS); one with same data except label “CSP25/SW4(2009)” (CASC: CASENT0914930); one with same data except 14.VI.2009, label “CSP25/NE5(2009)” (IZAS); one with same data except 29°12'53"N / 118°8'5"E, 366 m asl, label “CSP24/NW4(2009)” (IZAS); one with same data except 29°12'53"N / 118°8'5"E, 366 m asl, label “CSP24/SW4(2009)” (CASC: CASENT0914929); one with same data except 29°14'58"N / 118°7'19"E, 620 m asl, 26.VI.2009, label “CSP12/NE6(2009)” (ZMBH); one with same data except 29°14'47"N / 118°6'58"E, 402 m asl, 29.VIII.2009, label “CSP13/NW10(2009)” (IZAS).

##### Distribution.

**Figures 6.**
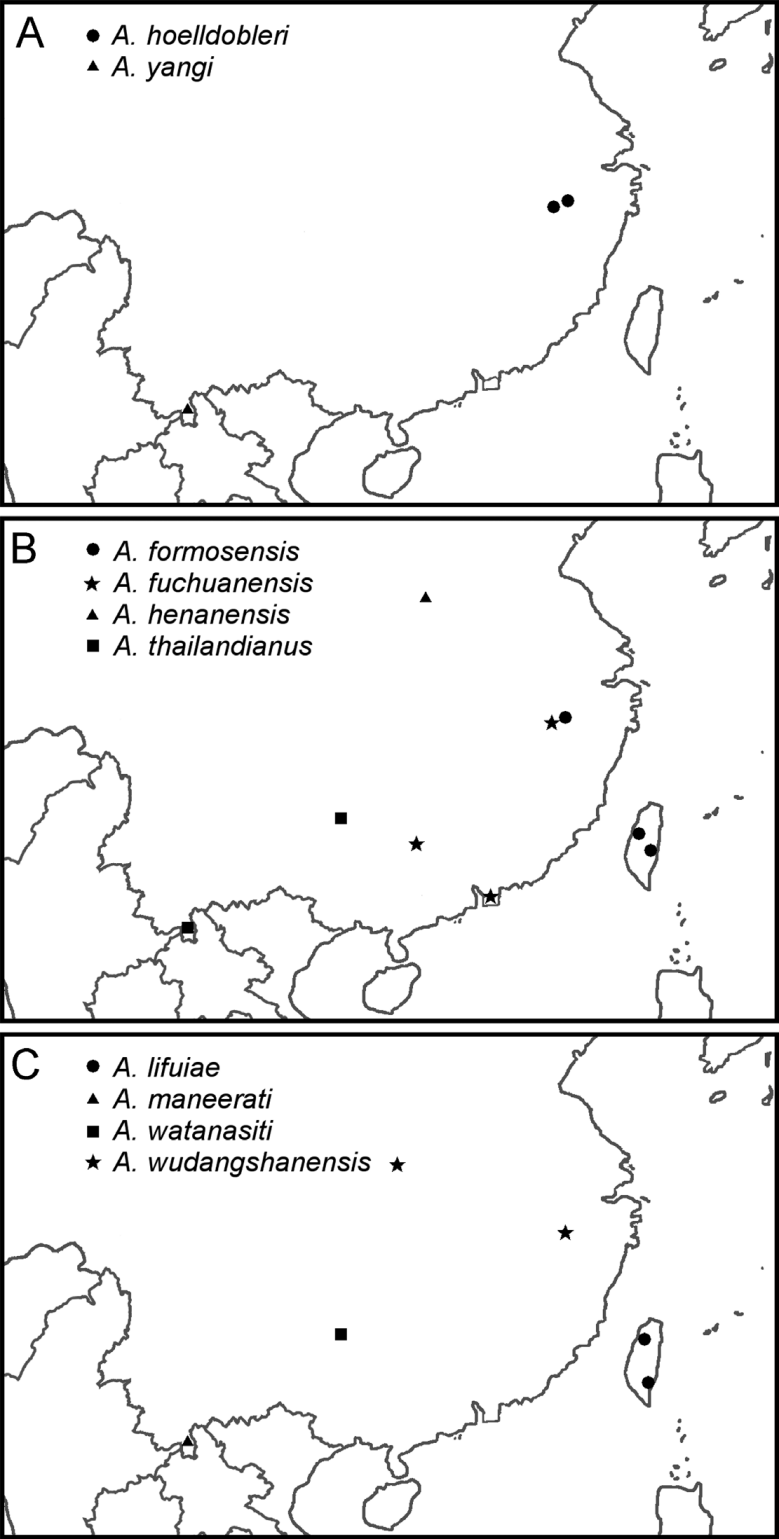
Distribution of the *Aenictus ceylonicus* group species in China. **A** *Aenictus hoelldobleri* sp. n. and *Aenictus yangi* **B** *Aenictus formosensis*, *Aenictus fuchuanensis*, *Aenictus henanensis*, and *Aenictus thailandianus* **C** *Aenictus lifuiae*, *Aenictus maneerati*, *Aenictus watanasiti*, and *Aenictus wudangshanensis*.

South-east China, provinces Zhejiang and Jiangxi (Fig. 6A).

##### Ecology.

The species is so far known to inhabit secondary mixed evergreen broad-leaved forests at mid elevations (ca. 300-620 m) where it occurs from young to old successional stages (referred to as “*Aenictus* (*ceylonicus* group) sp. CN02” in Staab et al. 2014, where more detailed biological information on the habitat can be found). Workers of a foraging column from one colony (MS1647) were observed during daytime (approximately 3pm) to carry pupae of *Technomyrmex* sp. and ant larvae on the ground. Thus, it is most likely that *Aenictus hoelldobleri* has a similar life history as other *Aenictus ceylonicus* group species by living and foraging on the ground and by being a specialized predator of other small ants.

##### Remarks.

*Aenictus hoelldobleri* is most similar to *Aenictus henanensis* Li & Wang, 2005 and *Aenictus wudangshanensis*, two species that also inhabit subtropical broad-leaved forests in China. *Aenictus hoelldobleri* can easily be distinguished from both species by the shape of the subpetiolar process, which is weakly developed and rounded in *Aenictus henanensis* (characters for *Aenictus hoelldobleri* are given in brackets: rectangular- trapezoidal, with a thin lamellae on the ventral outline) and rectangular with a very acute median apex that faces downwards in *Aenictus wudangshanensis*. Furthermore, *Aenictus henanensis* has the dorsum of the petiolar node smooth and shiny (finely reticulate) and lacks long, standing hairs on the dorsum of the head (longest hairs 0.15-0.20 mm). *Aenictus wudangshanensis* also has the mandible in total with 9 teeth/denticles (6-7). The three afore discussed species share with *Aenictus thailandianus*, *Aenictus lifuiae* Terayama, 1984, and *Aenictus yangi* Liu, Hita Garcia, Peng & Economo, 2015 the mandible with six or more teeth/denticles and the relatively small gap between the closed mandibles and the anterior clypeal margin. *Aenictus hoelldobleri* can be separated from *Aenictus thailandianus* by the sculpture of the dorsa of promesonotum and postpetiole, which are in *Aenictus thailandianus* entirely punctate and not shiny (smooth and shiny, at most very finely and superficially reticulate but still smooth and shiny). *Aenictus lifuiae* and *Aenictus yangi* differ from *Aenictus hoelldobleri* by having the dorsum of the mesonotum and the dorsum of the petiole entirely smooth and shiny (finely reticulate). Furthermore, the legs of *Aenictus lifuiae* are smooth and shiny (legs weakly punctate, most strongly on tibiae, coxae smooth and shiny) and the dentition of the mandible differs by having a large acute apical tooth followed by a series of 6-7 denticles of two sizes, the larger alternating with 1-2 smaller (large acute apical tooth, followed by a medium-sized subapical tooth, 3-4 minute denticles and a medium-sized basal tooth). The dentition of the mandible can also be used to separate *Aenictus hoelldobleri* from *Aenictus yangi*, in which the large acute apical tooth is followed by the medium-sized subapical tooth, one denticle, one medium sized tooth, two denticles, and the medium-sized basal tooth. Also, the maximum width of the gap between the anterior clypeal margin and the closed mandibles is in *Aenictus yangi* at least about as broad as the maximum width of the mandibles (gap clearly smaller than maximum width of mandible).

### New records of *Aenictus ceylonicus* group species from China

#### Aenictus formosensis

Taxon classificationAnimaliaHymenopteraFormicidae

Forel

Figs 2B , 4E

##### Non-type material examined.

Four workers from CHINA, Zhejiang Province, Gutianshan National Nature Reserve, ca. 30 km NW of Kaihua, 29°14'28"N / 118°6'37"E, 413 m asl, 30.VII.2008, pitfall trap in secondary mixed evergreen broad-leaved forest, leg. Andreas Schuldt, label: “CSP8/SE” (1 each in CASC: CASENT0914928 and IZAS).

##### Distribution.

Taiwan, Zhejiang (Fig. 6B).

##### Remarks.

This is the first record of *Aenictus formosensis* from the Chinese mainland. *Aenictus formosensis* has been described and illustrated in detail by Jaitrong and Yamane (2013, therein fig. 7A–C), who revived the species from synonymy under *Aenictus ceylonicus*. The four examined specimens collected in the Gutianshan National Nature Reserve agree very well with the material from Taiwan illustrated in Jaitrong and Yamane (2013) except that in one specimen the faces and the dorsum of the pronotum are very superficially reticulate but still shiny.

#### Aenictus fuchuanensis

Taxon classificationAnimaliaHymenopteraFormicidae

Zhou

Figs 2C , 4F

##### Non-type material examined.

Seven workers from CHINA, Jiangxi Province, near the village Xingangshan, ca. 15 km SE of Wuyuan, 29°5'21"N / 117°55'43"E, 136 m asl, 29.V.2013, hand collection on ground in an early successional tree plantation, leg. Michael Staab, label “MS1422” (1 each in CASC: CASENT0914926, IZAS, and ZMBH).

##### Distribution.

Guangxi, Hong Kong, Jiangxi (Fig. 6B); Cambodia, Laos, Thailand, Vietnam.

##### Remarks.

*Aenictus fuchuanesis* has been described and illustrated in detail by Jaitrong and Yamane (2013, therein fig. 8A–C), who extended the original description from Zhou (2001, therein figs 74–75). The seven examined specimens from the North-East of Jiangxi province agree in all aspects with the descriptions of Zhou (2001) and Jaitrong and Yamane (2013). This is so far the northernmost record of *Aenictus fuchuanensis*. Notably, the species was collected in an experimental tree plantation (see Bruelheide et al. 2014) that was planted four years prior and at the time of collection still had an open character with a maximum tree height of 3 m and abundant patches of bare soil. Hence, *Aenictus fuchuanensis* may be able to inhabit more open landscapes and not be restricted to forests, which may explain the relatively wide distribution of the species, which occurs from south Thailand to south-east China.

#### Aenictus thailandianus

Taxon classificationAnimaliaHymenopteraFormicidae

Terayama & Kubota

Figs 3A , 4A

##### Non-type material examined.

Three workers from CHINA, Guizhou Province, Leigongshan, 6.VII.1988, leg. Minsheng Wang; original label in Chinese “贵州雷公山 / 1988.VII.6 /王敏生 /中科院动物所“; (in IZAS: IOZ(E)1379709, all three workers on a single pin).

##### Distribution.

Guizhou, Yunnan (Fig. 6B); North Thailand, North Vietnam.

##### Remarks.

The three specimens of *Aenictus thailandianus* from Leigong Moutain, Guizhou Province, agree well with the original description of Terayama and Kubota (1993, therein figs 11–13) and the additional descriptions and illustrations of Jaitrong and Yamane (2013, therein fig. 17A–D). This is the so far northernmost record of *Aenictus thailandianus*, which has in China only been recorded from Yunnan (Liu et al. 2015a).

#### Aenictus watanasiti

Taxon classificationAnimaliaHymenopteraFormicidae

Jaitrong & Yamane

Fig. 1C

##### Non-type material examined.

Three workers from CHINA, Guizhou Province, Leigongshan, 6.VII.1988, leg. Minsheng Wang; original label in Chinese “贵州雷公山 / 1988.VII.6 /王敏生 /中科院动物所“; (in IZAS: IOZ(E)1379710, all three workers on a single pin).

##### Distribution.

Guizhou (Fig. 6C); Thailand, North Vietnam.

##### Remarks.

The three specimens of *Aenictus watanasiti* from Leigong Moutain, Guizhou Province, agree very well with the original description and the illustrations of Jaitrong and Yamane (2013, therein fig. 18A–D). This is the so far northernmost record of *Aenictus watanasiti* and the first record from China.

#### Aenictus wudangshanensis

Taxon classificationAnimaliaHymenopteraFormicidae

Wang

Fig. 4D

##### Non-type material examined.

Four workers from CHINA, Zhejiang Province, Gutianshan National Nature Reserve, ca. 30 km NW of Kaihua, 29°15'18"N / 118°8'51"E, 880 m asl, 25.VI.2009, pitfall trap in secondary mixed evergreen broad-leaved forest, leg. Andreas Schuldt, label: “CSP6/SE6(2009)” (1 each in CASC: CASENT0914927 and IZAS).

##### Distribution.

Hubei, Zhejiang (Fig. 6C).

##### Remarks.

So far *Aenictus wudangshanensis* has been known only from the type series collected in the Wudangshan Nature Reserve, Hubei Province. The four specimens from the Gutianshan National Nature Reserve agree very well with the original description of Wang (2006, therein figs 1.2). Like the type series, the specimens were collected at mid elevation in an evergreen broad-leaved forest and *Aenictus wudangshanensis* may be restricted to this habitat type.

## Discussion

The genus *Aenictus* with its type-species *Aenictus ambiguus* Shuckard, 1840 was originally established and described based on the male caste. In the Chinese *Aenictus* fauna there are eight species and subspecies only known from males (Guénard and Dunn 2012, AntCat 2015); among the Chinese *Aenictus ceylonicus* group species listed in the present paper, the male is known only for *Aenictus lifuiae* (see figs 5–13 in Terayama 1984). Male-worker associations are as yet unclear for other species. It is thus not impossible that the species described here as new corresponds to one of the already described male-based taxa. To avoid unnecessary synonyms it may be more appropriate to refrain from new descriptions of *Aenictus* species until male-worker combinations are better understood, for example by applying barcoding approaches (see e.g. Huemer et al. 2014). Not describing new species causes, however, a different problem. Only published species names will be included in species lists and be available for further studies (see also Wilson 1964 and Jaitrong and Yamane 2013 for a more detailed discussion), including conservation planning. Weighing these arguments, it was decided to describe the new species. Barcoding data on species level would also help to further strengthen the taxonomic concept in the genus *Aenictus*. So far, species groups and single species (including the species newly described here) are solely based on sometimes rather minor differences in morphological characters such as the shape of the subpetiolar process. As long as no genetic data are available it may be argued that such differences are a form of intraspecific variation, as interpreted by Wilson (1964).

Most *Aenictus* species are largely restricted to forests. Unfortunately, forests in China and elsewhere in Asia have been and are still continuously cleared and transformed into agriculture or tree plantations (e.g. López-Pujol et al. 2006, Hansen et al. 2013). Being top predators of the leaf-litter food web characterized by low dispersal abilities and consequently rather limited distribution ranges (Gotwald 1995, Jaitrong and Yamane 2013), *Aenictus* species are expected to be directly negatively affected by forest loss and anthropogenic land use as shown by Matsumoto et al. (2009). Hence, the ongoing forest conversion may sooner or later endanger ant species with a specialized life-history such as *Aenictus*.

There are many records of *Aenictus ceylonicus* from south and east China (listed in Guénard and Dunn 2012). This species was formerly thought to be widely distributed from India to Australia (Wilson 1964). However, more recent work has shown that the ‘true’ *Aenictus ceylonicus* is almost certainly restricted to India and Sri Lanka (Shattuck 2008, Jaitrong and Yamane 2013). Thus, all Chinese records of this species have to be considered as highly doubtful and should be critically reevaluated. It is likely that these records in fact refer to one or several of the species (such as *Aenictus formosensis*) treated in Jaitrong and Yamane (2013) and in the present paper. Recently, *Aenictus formosensis* was revived from synonymy under *Aenictus ceylonicus* by Jaitrong and Yamane (2013) and is here reported for the first time from the Chinese mainland. I hope that the key to the Chinese *Aenictus ceylonicus*-group species presented here may assist the necessary clarifications and reevaluations.

The diversity center for the *Aenictus ceylonicus* group seems to be in continental South-East Asia (Jaitrong and Yamane 2013). There are several species such as *Aenictus brevipodus* Jaitrong & Yamane, 2013, which have been described from North Vietnam, close to the Chinese border. It is very likely that most of the *Aenictus ceylonicus*-group species that are so far only known from the North of Vietnam or northern Thailand also extend their range into Southern China, as the finding of *Aenictus thailandianus* and *Aenictus watanasiti* in Guizhou Province demonstrates. Further sampling in the highly endangered tropical and subtropical forests of China is necessary to fully capture the diversity and distribution ranges of ants, including *Aenictus*. Given the limited distribution of most *Aenictus* species and the generally understudied Chinese ant fauna (Guénard and Dunn 2012, Liu et al. 2015a) it is likely that such sampling will also reveal further, as yet undescribed species.

## Supplementary Material

XML Treatment for Aenictus hoelldobleri

XML Treatment for Aenictus formosensis

XML Treatment for Aenictus fuchuanensis

XML Treatment for Aenictus thailandianus

XML Treatment for Aenictus watanasiti

XML Treatment for Aenictus wudangshanensis
